# Serum and Follicular Fluid Thiol/Disulfide Homeostasis in Diminished Ovarian Reserve Patients Undergoing In Vitro Fertilization/Intracytoplasmic Sperm Injection Treatment

**DOI:** 10.7759/cureus.35476

**Published:** 2023-02-26

**Authors:** Kadriye Erdoğan, Nazlı Şanlıer, Emine Utlu, Huri Güvey, İnci Kahyaoğlu, Salim Neşelioğlu, Özcan Erel, Serra Akar, Yaprak Engin-Üstün

**Affiliations:** 1 Obstetrics and Gynaecology, Etlik Zübeyde Hanım Women's Health Training and Research Hospital, Ankara, TUR; 2 Obstetrics and Gynaecology, City Hospital, Ankara, TUR; 3 Histopathology, Etlik Zübeyde Hanım Women's Health Training and Research Hospital, Ankara, TUR; 4 Obstetrics and Gynaecology, Kütahya Parkhayat Hospital, Kütahya, TUR; 5 Biochemistry, City Hospital, Ankara, TUR; 6 Biochemistry, Yıldırım Beyazit University, Ankara, TUR; 7 Obstetrics and Gynaecology, University of Health Sciences Etlik Zubeyde Hanim Women’s Health Training and Research Hospital, Ankara, TUR; 8 Obstetrics and Gynaecology, Zekai Tahir Burak Women's Health Research and Education Hospital, Ankara, TUR

**Keywords:** antioxidant supplementation, under 35 years of age, diminished ovarian reserve, thiol/disulphide homeostasis parameters, serum and follicular fluid

## Abstract

Introduction: The etiologies of diminished ovarian reserve (DOR) are still poorly understood, and many factors such as age, autoimmunity, genetics, idiopathicity, iatrogenesis, and oxidative stress (OS) play a role. Oxidative cellular damage increases following reactive oxygen species (ROS)-induced aging. This is the first study to evaluate the serum and follicular fluid (FF) thiol/disulfide homeostasis in patients under 35 years of age with DOR undergoing in vitro fertilization (IVF)/intracytoplasmic sperm injection (ICSI).

Methods: In this study, DOR was defined by the Poseidon criteria, and Poseidon group 3 women were selected as the study group (n = 40). The control group was composed of patients with the diagnosis of mild-moderate male factor infertility (n = 30).

Results: The FF and serum native and total thiol levels, the markers of the antioxidant system, were significantly decreased in the DOR group compared with the control group (p = 0.021) (p = 0.037) (p = 0.029) (p = 0.04). On the other hand, we found no significant differences in the oxidant parameters between the groups (p > 0.05).

Conclusions: An intrinsic deficiency of antioxidants can play an important role in the etiology of DOR. The dietary addition of antioxidants could be beneficial in DOR patients.

## Introduction

Diminished ovarian reserve (DOR) is defined as a decrease in oocyte number and quality and indicates a poor response to ovarian stimulation compared with women of comparable age [1]. The poor response has been defined by Bologna or Poseidon criteria [2]. The incidence of DOR in infertile women is between 6% and 64% in different age groups [3]. However, the etiologies of DOR are still poorly understood and many factors such as age, autoimmunity, genetics, idiopathicity, iatrogenesis, and oxidative stress (OS) play a role [4].

Although physiological levels of reactive oxygen species (ROS) are required for oocyte maturation, physiological follicular atresia, ovulation, and fertilization, the overabundance of ROS impairs normal reproductive functioning [5]. Thiols are antioxidants containing sulfhydryl groups that act as the electron acceptors of ROS. By the neutralizing actions of thiols, the oxidants are converted to less toxic products and disulfide molecules are formed. The thiol/disulfide pools play a vital role in intracellular redox homeostasis, which is critical for antioxidant defenses [6]. There is a balance between thiol and disulfide. The shift in the balance in favor of oxidants defines OS. The thiol/disulfide equilibrium is an indicator of OS [7].

The follicular fluid (FF) is produced by granulosa cells [8] and is composed of metabolic products, steroid hormones, polysaccharides, proteins, ROS, and antioxidants that affect folliculogenesis. Enzymatic antioxidant pathways are needed to keep the ROS levels of the FF at an appropriate level. Variations in the microenvironment of the FF may impair oocyte quality and maturation [9]. Oxidative cellular damage increases following ROS-induced aging [10].

The exclusion of the age factor might be beneficial for the determination of more precise etiologies that affect the reproductive pathways. Therefore, the objective of this study was to investigate the serum and FF thiol/disulfide homeostasis in patients under 35 years of age with DOR who underwent in vitro fertilization (IVF)/intracytoplasmic sperm injection (ICSI) treatment.

## Materials and methods

Study design

**Figure 1:**
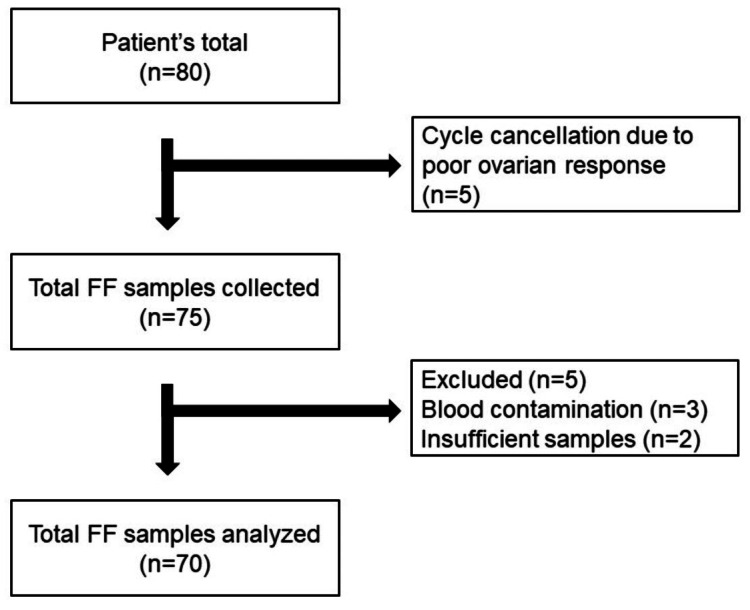
Study Flow Chart FF, follicular fluid.

A prospective study was undertaken at the IVF clinic of a tertiary referral center. A total of 80 patients were initially selected for the study. However, cycle cancellation due to poor ovarian reserve in five patients resulted in the collection of FF samples from 75 patients. Out of the 75 samples, five samples were excluded due to blood contamination in three patients and insufficient sample collection in two patients. Finally, the study included a total of 70 women, aged 18-35 years. The study flow chart is reported in Figure 1. The Local Ethical Committee (06.04.2022 2022/50) approved the study protocol. In this study, DOR was defined by the Poseidon criteria, and Poseidon group 3 women were selected as the study group (n = 40) (3). The control group was composed of patients with the diagnosis of mild-moderate male factor infertility (n = 30).

Patients with a body mass index (BMI) >35 kg/m^2^, age <18 and >35 years, a history of smoking, acute infection (within 14 days), systemic, endocrine, or inflammatory diseases, use of any medications including vitamin supplementation, patients who received mild or natural cycle protocols, freeze-thawed cycles, more than one embryo transfer, and couples with severe male infertility such as azoospermia and severe oligoasthenospermia were excluded.

Age, BMI, partner’s age, number of cycles, and duration of infertility were recorded. Day 3 (D3) basal serum hormone levels, anti-mullerian hormone (AMH) levels, antral follicle count, number of retrieved and metaphase II (MII) oocytes, Grade 1-2-3 embryos [11], blastocyst quality scoring (BQS) [12], day of embryo transfer, clinical pregnancy after fresh transfer cycle, and serum and follicular thiol/disulfide homeostasis parameters were measured.

Controlled ovarian stimulation and collection of FF and blood samples

A standardized gonadotropin-releasing hormone long agonist protocol was used in all patients [13]. A 250 μg dose of human chorionic gonadotrophin (Ovitrelle®, Merck, Germany) was given when at least three follicles were 17-18 mm in diameter. Oocytes were retrieved 34-36 h later. Oocytes were incubated as cumulus complex at 37°C, 5% CO_2_, and 5% O_2_ for 2 h before denudation for ICSI. Denudation was completed by both hyaluronidase (Hyase 10X, Vitrolife, Sweden) and the mechanical technique. Fertilization was confirmed by the presence of two pronuclei 18-20 h after the ICSI. One-step culture protocol (G-TL, Vitrolife, Sweden) was used for the embryo culture under oil at 37°C, 5% CO_2_, and 5% O_2_ incubator (Miri, ESCO Medical, Turkey) conditions [11]. Embryos were graded according to the morphological criteria of Gardner and Schoolcraft [14], and BQS was performed according to the criteria reported by Rehman et al. [12]. Cleavage-stage embryos or blastocysts were transported to the uterine cavity. Luteal-phase sustenance was provided with intramuscular progesterone (Progestan®, Koçak Pharma, Turkey) and/or oral dydrogesterone (Duphaston®, Abbott, Turkey) until 12 weeks gestational age in all patients. Clinical pregnancy was recorded with the observation of a gestational sac by transvaginal ultrasound.

For each patient, a single follicle measuring between 18 and 22 mm was chosen for the collection of FF samples. The chosen FF samples without macroscopic blood contamination were subjected to centrifugation for 10 min at 1200 *g* to remove cellular components. Blood was taken immediately prior to the retrieval of oocytes and the sera were separated following centrifugation at 1200 *g* for 10 min. The serum and FF samples were kept at -80 degrees until thiol/disulfide homeostasis values were examined.

Analysis of thiol/disulfide homeostasis values

Automated spectrophotometry was used for thiol/disulfide homeostasis tests as detailed by Erel & Neselioglu. Briefly, upon the reduction of disulfide bonds to free thiol groups with sodium borohydride, left-over sodium borohydride was cleared with formaldehyde to prevent the reduction of 5,5’-dithiobis-2-nitrobenzoic acid (DTNB). Following the reaction with DTNB, all of the thiol groups including both the reduced and native thiol groups were determined. Serum native thiol level is measured directly using Ellman reagent without performing these reduction processes. The dynamic disulfide value was obtained by halving the difference between the total thiols and the native thiols. Once the native and total thiols were calculated, disulfide values and disulfide/native thiol percent ratios (SS/SH %) were determined. Percent coefficient variation (%CV) was 4 (X̅= 29.12 and σX = 1.2) for high levels, 5 (X̅=16.03 and σX = 0.79) for medium levels, and 13 (X̅= 7.15 and σX = 0.98) for low levels [15].

Statistical analysis

Descriptive analyses were expressed using the mean and standard deviation (mean ± SD) for parametrically distributed variables and the median (minimum-maximum) for non-parametrically distributed variables. Parametric and non-parametric variables between groups were compared using the independent-samples t-test and Mann-Whitney U test, respectively. Pearson’s correlation was used to investigate the potential association between variables. A p-value <0.05 was accepted to be statistically significant. All analyses were performed by SPSS 20.0 (IBM Corp., Armonk, NY, USA).

## Results

Totally 70 patients were included in the study. The comparisons between groups did not reveal any significant differences in age, partner’s age, BMI, D3 luteinizing hormone (LH), number of cycles, and duration of infertility (p > 0.05) (Table 1). The AMH levels and total antral follicle count were significantly lower in DOR patients (p < 0.001) whereas the D3 follicle-stimulating hormone (FSH) and estradiol (E2) were significantly elevated in DOR patients in comparison to the control group (p < 0.001) (p = 0.037) (Table 1).

**Table 1:** Demographic characteristics and laboratory data *Values are mean±standard deviation; **values are median (min-max); ***p-value of less than 0.05 was considered to be statistically significant. BMI, body mass index; AMH, antimullerian hormone; D3, day 3; FSH, follicle-stimulating hormone; LH, luteinizing hormone; E2, estradiol; AF, antral follicle.

Parameters	Control Group (n=30)	Study Group (n=40)	p-Value
Age (years)*	30.21 ± 3.72	30.4 ± 3.56	0.584
Partner’s age (years)*	31.56 ± 6.35	30.81 ± 6.92	0.326
BMI (kg/m^2)**^	24.85 (18.5-34.3)	25 (19-33.8)	0.521
AMH (ng/mL)**	2.85 (1.4-3.4)	0.55 (0.03-1.05)	<0.001***
D3 FSH (mIU/mL)*	6.28 ± 1.06	9.14 ± 3.95	<0.001***
D3 LH (mIU/mL)*	6.82 ± 2.3	6.73 ± 2.67	0.578
D3 E2 (pg/mL)*	34.41 ± 9.6	42.31 ± 21.01	0.037***
Total AF count**	13 (8-16)	4 (0-9)	<0.001***
Number of cycles**	1 (1-3)	1 (1-5)	0.582
Duration of infertility (months)**	48 (18-144)	42 (3-228)	0.553

No difference of significant sort was figured out either in the number of Grade 3 embryos, day of embryo transfer, and clinical pregnancy rates between the two groups (p > 0.05) (Table 2). The number of retrieved oocytes, MII oocytes, Grade 1-2 embryos, and the BQS were significantly lower in DOR patients than in the control group (p < 0.001) (p < 0.001) (p = 0.039) (p = 0.023) (p = 0.038) (Table 2).

**Table 2:** Outcomes of in vitro fertilization cycles *Values are mean ± standard deviation; **values are median (min-max); ***p-value of less than 0.05 was considered to be statistically significant. MII, metaphase II; BQS, blastocyst quality scoring.

Parameters	Control Group (n = 30)	Study Group (n = 40)	p-Value
Retrieved oocyte count*	9.23 ± 5.53	4.27 ± 3.1	<0.001***
MII oocyte count*	7.54 ± 4.74	3.18 ± 2.4	<0.001***
Number of Grade 1 embryo**	1 (0-5)	1 (0-1)	0.039***
Number of Grade 2 embryos**	1 (0-2)	0 (0-2)	0.023***
Number of Grade 3 embryos**	1 (0-3)	0 (0-1)	0.141
Day of embryo transfer**	5 (2-5)	3 (3-5)	0.057
BQS*	32.11 ± 11.82	16.50 ± 4.94	0.038***
Clinical pregnancy rate (%)	46.2%	40%	0.552

Significant differences were observed concerning antioxidant levels: the FF native and total thiol and serum native and total thiol levels were significantly decreased in the DOR group compared to the control group (p = 0.021) (p = 0.029) (p = 0.037) (p = 0.04) (Table 3).

**Table 3:** Follicular fluid and serum thiol/disulfide homeostasis parameters between the groups *p-Value of less than 0.05 was considered to be statistically significant. FFNT, follicular fluid native thiol; FFTT, follicular fluid total thiol; FFD, follicular fluid disulfide; FFD/NT, follicular fluid disulfide/native thiol; SNT, serum native thiol; STT, serum total thiol; SD, serum disulfide; SD/NT, serum disulfide/native thiol.

Parameters	Control Group (n = 30)	Study Group (n = 40)	p-Value
FFNT (µmol/L)	433.03 ± 42.1	399.67 ± 48.27	0.021*
FFTT (µmol/L)	481.18 ± 42.75	448.14 ± 52.02	0.029*
FFD (µmol/L)	24.08 ± 3.11	24.23 ± 5.23	0.727
FFD/NT (%)	5.61 ± 0.88	6.11 ± 1.4	0.129
SNT (µmol/L)	494.62 ± 29.99	467.96 ± 46.24	0.037*
STT (µmol/L)	554.23 ± 32.72	524.15 ± 50.14	0.04*
SD (µmol/L)	29.81 ± 4.46	28.09 ± 5.56	0.585
SD/NT (%)	6.04 ± 0.92	6.04 ± 1.18	0.6

We found no significant differences in the oxidant parameters (the FF and serum disulfide level and the ratios of FF and serum disulfide/native thiols) between the groups (p > 0.05) (Table 3). We observed a positive correlation between serum and FF antioxidant-oxidant parameters (r = 0.389, p = 0.009) (r = 0.413, p = 0.005) (r = 0.376, p = 0.012) (r = 0.341, p = 0.023) (Table 4).

**Table 4:** Correlation analysis of serum and follicular fluid thiol/disulfide homeostasis parameters *p-Value of less than 0.05 was considered to be statistically significant. FFNT, follicular fluid native thiol; FFTT, follicular fluid total thiol; FFD, follicular fluid disulfide; FFD/NT, follicular fluid disulfide/native thiol; SNT, serum native thiol; STT, serum total thiol; SD, serum disulfide; SD/NT, serum disulfide/native thiol.

Parameters		SNT	STT	SD	SD/NT
FFNT (µmol/L)	r	0.389	0.42	0.118	-0.001
	p	0.009*	0.004*	0.445	0.997
FFTT (µmol/L)	r	0.378	0.413	0.174	0.057
	p	0.011*	0.005*	0.26	0.712
FFD (µmol/L)	r	0.077	0.111	0.376	0.358
	p	0.62	0.469	0.012*	0.017*
FFD/NT (%)	r	-0.142	-0.123	0.303	0.341
	p	0.358	0.427	0.046*	0.023*

A direct correlation was found between BQS and the FF and serum native thiol levels and the FF and serum total thiol levels (r = 0.733, p = 0.016) (r = 0.837, p = 0.005) (r = 0.703, p = 0.023) (r = 0.875, p = 0.002). In contrast, the FF disulfide and the ratios of FF disulfide/native thiols showed a negative correlation with BQS (r = -0.698, p = 0.025) (r = -0.724, p = 0.018). Also, the serum disulfide level and the ratios of serum disulfide/native thiols did not show any correlation with BQS (p > 0.05) (Table 5).

**Table 5:** Correlation analyses between BQS and serum and follicular fluid thiol/disulfide homeostasis parameters *p-Value of less than 0.05 was considered to be statistically significant. FFNT, follicular fluid native thiol; FFTT, follicular fluid total thiol; FFD, follicular fluid disulfide; NT, native thiol; SNT, serum native thiol; STT, serum total thiol; SD, serum disulfide; BQS, blastocyst quality scoring.

Parameters		FFNT	FFTT	FFD	FFD/NT	SNT	STT	SD	SD/NT
BQS	r	0.733	0.703	-0.698	-0.724	0.837	0.875	0.274	-0.287
	p	0.016*	0.023*	0.025*	0.018*	0.005*	0.002*	0.475	0.454

## Discussion

To our knowledge, this study is the first to investigate serum and FF thiol/disulfide homeostasis in DOR patients undergoing IVF/ICSI treatment to evaluate OS impact. OS is critical in the development of age-related cellular damage. Debbarh H et al. reported that the age cutoff for the increase in OS parameters of FF is 37 [10]. In this study, we excluded the age factor by selecting patients under 35 years of age. In our study, we observed that the serum and FF total and native thiol levels, markers of the antioxidant system, were decreased in DOR patients. On the other hand, the serum and FF disulfide levels, and the serum and FF disulfide/native thiol ratios, which reflect the status of OS, were not higher in DOR patients in comparison to the control group. Also, a direct relationship was found between serum and FF thiol/disulfide homeostasis. It is noteworthy to highlight here that the antioxidant levels were decreased without an increase in oxidant parameters in the serum and FF of patients with DOR. It can be concluded that the decrease in antioxidants solely might be one of the causes of DOR. Thus, the dietary addition of antioxidants could be beneficial in DOR patients. The pathophysiology of DOR is not fully understood; OS and mitochondrial dysfunction are mostly blamed. During the development of oocytes, excessive ROS are produced in the mitochondria. The inadequacy in the scavenging of ROS by antioxidant defenses results in mitochondrial dysfunction and OS [16]. Antioxidant supplementation is recommended to improve reproductive outcomes in DOR patients to regulate mitochondrial function and neutralize OS. Many different antioxidants such as vitamins C, A, and E, coenzyme Q10, Nobiletin, cysteamine, melatonin, and L-arginine were used [17]. It is not clear which antioxidants are more effective. The most popular one is coenzyme Q10 which is a critical component of the inner mitochondrial membrane. Xu et al. found that the reproductive outcomes of young women with DOR were improved by the oral administration of coenzyme Q10 [18].

Based on the literature review, FF thiol/disulfide homeostasis was evaluated only in polycystic ovary syndrome (PCOS)-related female infertility undergoing IVF/ICSI treatment. Tola et al. revealed that the FF native thiol levels and the FF native thiol/total thiol ratios were lower in women with PCOS, whereas the FF disulfide levels and the FF disulfide/native thiol and disulfide/total thiol ratios were increased [19]. Contrarily, Yildirim et al. showed increased serum thiol levels and decreased disulfide levels in PCOS patients, and the discrepancy between the results might be due to the type of samples as FF used in a study by Tola et al. [20]. In addition, Özdemir et al. demonstrated that the serum thiol/disulfide homeostasis did not differ among patients with male factor infertility, unexplained infertility, and DOR [21].

In line with the literature, we revealed that the value of AMH was lower in DOR patients, whereas the D3 FSH and E2 were higher. The production of inhibin B and AMH by the granulosa cells is reduced with the diminishing follicular pool. A decline in the secretion of inhibin B results in a quicker and earlier FSH elevation in the follicular phase. Additionally, quicker folliculogenesis may lead to an elevation of E2 levels in DOR patients [22].

Consistent with previous research, we showed that the number of retrieved and MII oocytes and the quality of embryos were lower in DOR patients [23,24]. We observed that the BQS had a positive correlation with serum and FF antioxidant parameters. On the other hand, BQS showed a negative correlation with FF oxidant parameters and did not show any correlation with serum oxidants. In contrast, Özdemir et al. revealed that the increased serum disulfide levels had a positive correlation with MII oocytes and they explained this finding by the necessity of a small amount of OS for oocyte development [21]. In folliculogenesis, the FF microenvironment was more pivotal than that of serum. Thus, oocyte quality might not be impaired by tolerable serum oxidant levels. Liu et al. showed that the OS markers were higher in both serum and the FF of PCOS patients, and these markers could be used for predicting embryo quality [25]. In a study by Artini et al., the FF drawn from fertilized oocytes contained lower proinflammatory cytokines than in unfertilized ones and the antioxidant potential had a direct correlation with the number of retrieved, mature, and fertilized oocytes [26]. Although OS was higher and BQS was lower in DOR patients, we found similar clinical pregnancy rates between both groups. This conflicting result was probably due to the limited number of participants in each group.

## Conclusions

In conclusion, the etiologies of DOR are still poorly understood and many factors are blamed. The exclusion of the age factor might be beneficial for the determination of more precise etiologies that affect the reproductive pathways. In this study, the antioxidant levels were decreased without an increase in oxidant parameters in the serum and FF of patients with DOR. An intrinsic deficiency of antioxidants can play an important role in the etiology of DOR. Thus, the dietary addition of antioxidants could be beneficial in DOR patients. Larger studies are required to confirm our results.
